# Maternal mid- and late-pregnancy distress and birth outcome: A causal model of the mediatory role of pregnancy-specific distress

**DOI:** 10.18502/ijrm.v17i8.4824

**Published:** 2019-09-03

**Authors:** Mahbobeh Faramarzi, Parvin Hassanjanzadeh, Soraya Khafri

**Affiliations:** ^1^Social Determinants of Health Research Center, Health Research Institute, Babol University of Medical Sciences Babol Iran.; ^2^Psychology Department, Ayatollah Amoli Branch, Islamic Azad University Amol Iran.; ^3^Infertility and Health Reproductive Research Center, Health Research Institute, Babol University of Medical Sciences Babol Iran.

**Keywords:** Birth outcomes, Pregnancy, Distress, Anxiety, Depression

## Abstract

**Background:**

There is lack of information about the effect of general distress and pregnancy-specific distress in mid- and late-pregnancy separately on neonatal outcome.

**Objective:**

The aim of this study was to assess the effects of mid-maternal distress on late-maternal distress and birth outcomes with a causal model of relationships among general distress and pregnancy-specific distress.

**Materials and Methods:**

In this longitudinal descriptive study, 100 low-risk pregnant women participated. Participants completed three questionnaires at mid-pregnancy (13–26 wk) and at late pregnancy (27–40 wk). Pregnancy-general distress was assessed by the Perceived Stress Scale and the Hospital Anxiety Depression Scale. Pregnancy-specific distress was evaluated by the Prenatal Distress Questionnaire. The pregnant women were followed to after birth and neonatal outcome were assessed.

**Results:**

All total effect pathways were significant as predictors of birth outcomes (height, weight, and head circumference). Mid-pregnancy-specific distress had a significant relationship with late pregnancy-specific distress. However, mid-maternal distress was not related directly to birth outcomes. The effect of mid-maternal distress on birth outcomes was related indirectly to late-maternal distress. Both late general distress and late pregnancy-specific distress had direct negative effects on three indexes of birth outcome. The negative effect of late general-pregnancy distress and mid-pregnancy-specific distress on birth outcome was mediated through late pregnancy-specific distress.

**Conclusion:**

Both late pregnancy-general distress and pregnancy-specific distress have negative effects on birth outcomes. These findings support a role for negative effect as mediating the relationship between late pregnancy-specific distress and birth outcomes.

## 1 Introduction

Maternal distress, the experience of pregnancy-general distress (stress, anxiety, and depression) and pregnancy-specific distress during pregnancy and delivery is common (1, 2). Maternal distress is associated with negative consequences on maternal and fetal health. Evidence suggests that antenatal maternal distress increases the risk of spontaneous abortion, preterm delivery, and hypertension (3). Maternal distress during pregnancy was found to adversely influence the growth of the fetus (4). Although evidence supports the effect of psychological factors on maternal stress, suitable adaptation to stress may influence birth outcomes (5–7).

There is the controversy that maternal distress is related to the time of pregnancy. A study reported that depressive symptoms and maternal distress in early pregnancy where related to maternal distress during late pregnancy. (8). A study showed that both state and trait anxiety were not different among pregnant women in early-, mid-, and late pregnancy (9). Although some large cohort studies reported correlations between high levels of anxiety and depression during pregnancy and adverse birth outcomes (10), studies that explore the relationship between maternal distress in mid- and late study and effect on neonatal outcome have not been addressed sufficiently. A cohort study reported that maternal distress during pregnancy was a risk factor for childhood disease in the offspring (11).

**Figure 1:**
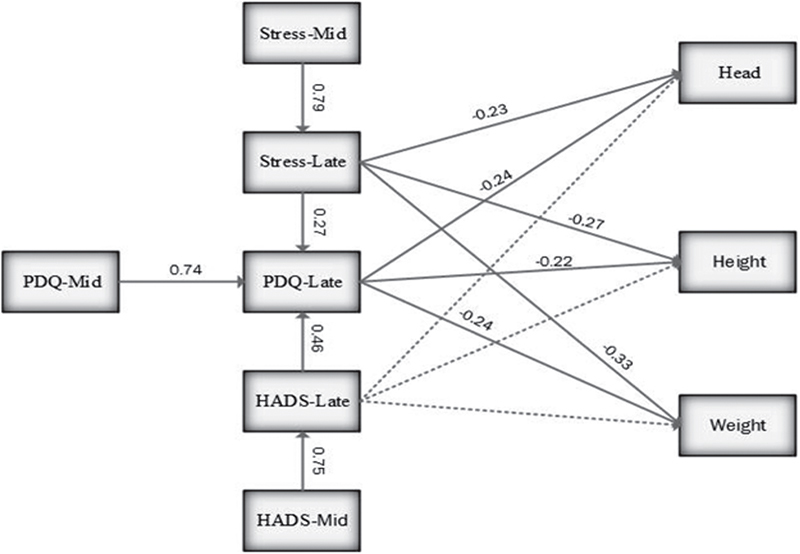
Final path model with standardized path coefficients of predictors of birth outcomes. Stress-Mid: perceived stress in mid-pregnancy. Stress-Late: perceived stress in late-pregnancy. PDQ: Prenatal Distress Questionnaire. PDQ-Mid: Prenatal Distress Questionnaire in mid-pregnancy. PDQ-Late: Prenatal Distress Questionnaire in late-pregnancy. HADS: Hospital Anxiety Depression Scale. HADS-Mid: Hospital Anxiety Depression Scale in mid-pregnancy. HADS-Late: Hospital Anxiety Depression Scale late-pregnancy

The aims of this study were to: 1) explore whether mid-maternal distress is related to birth outcome; 2) explore whether late-maternal distress is associated with birth outcome; 3) explore the association between mid-maternal distress and late-maternal distress; and 4) test the model depicted in Figure 1 of the proposed interrelationships - whether the effect of mid-maternal distress on birth outcome was mediated by late pregnancy-specific distress.

## 2 Materials and Methods

A prospective study was conducted in health centers of the Babol University of Medical Sciences from December 2014 to June 2015. Inclusion criteria were: pregnant women who had at least 5░yr of education, over the age of 18░yr, agreed to enter the study, a gestational age between 13 and 26 wk, and self-reported that they were healthy and at low risk of developing complications. The women who were classified as being at risk for pregnancy complications were excluded from the study. Convenience sampling was utilized to recruit the women based on the multi-stage cluster. Of the 115 eligible women who consented to participate in the study, 100 women continued the study through their third trimester.

Five midwives gave a brief explanation regarding the purpose of the study and how to fill in the questionnaires. Participants completed three questionnaires; the Hospital Anxiety Depression Scale (HADS), the Prenatal Distress Questionnaire (PDQ), and the Perceived Stress Scale (PSS-14) during antenatal care appointments at mid-pregnancy (13–26 wk) and late pregnancy (27–40 wk). Pregnancy-general distress was assessed by two questionnaires including PSS-14 and HADS. Pregnancy-specific distress was evaluated by one questionnaire; the PDQ. The women were followed to birth. After the child birth, a midwife gathered the medical records of newborns from the files, including height, weight, and head circumference of the neonates.

### 2.1 Measures

#### 2.1.1 Perceived Stress Scale (PSS-14)

It contains 14 items and assesses the public perceived stress in the past month. The responses of the PSS-14 are given on a five-point Likert scale ranging from 0 (never) to 4 (almost). PSS-14 has a possible range of 0–56. The higher points indicate higher perceived stress. In this study, the Cronbach’s alpha coefficient of the PsS-14 was 0.789.

#### 2.1.2 PDQ

It consisted of 12 items to assess pregnancy-specific distress. It has three reliable subscales; concerns about emotions and relationships, weight/body image, and birth and the baby. The responses of the PDQ were given on a five-point Likert scale ranging from 0 (not at all) to 4 (extremely). In this study, the Cronbach’s alpha coefficient of the PDQ was 0.754.

#### 2.1.3 HADS

Hospital Anxiety Depression Scale consists of 14 items (seven questions on anxiety and seven questions on depression) to screen the presence and severity of anxiety and depression. The score of each item ranged 0–3, therefore total score of anxiety or depression ranged 0–21. (12). In the present study, HADS had 0.783 Cronbach’s alpha coefficient.

### 2.2 Ethical consideraqtion

All aspects of the study were approved by the research committee of the Babol University of Medical Sciences (Code: IR.MUBABOL.REC.1397.029). All of the participants signed the informed consent form.

### 2.3 Statistical analysis

To investigate the relationship between neonatal outcomes and maternal distress, path analysis was done. The factors for path model were selected based on previous studies. The hypothesis was assumed from the literature that mid-anxiety/mid-depression affects birth outcomes both directly and indirectly (late-anxiety/depression). Mid-perceived stress affects birth outcomes both directly and indirectly (late-anxiety/depression and late-perceived stress). In addition, late pregnancy-specific stress mediates the relationship between late-anxiety/depression and late-perceived stress with the birth outcome. Therefore, the separate full model for each of the birth outcomes included more than eight nuisance parameters in addition to the interest parameters. The standard beta coefficient (B) obtained from the multiple regression model represented the correlation coefficient between variables; R^2^ (coefficient of determination) obtained from the regression model represented the efficiency of the model.

## 3 Results

About 68% of women’s age was under 30░yr and 90% of them were employed. The mean of maternal distress were increased in late, but this increase was statistically significant (p░<░0.001) (Table I). Figure 1 shows that the final model, illustrating the significant relationship between mid- and late-pregnancy distress. The significant pathways of the model for birth outcomes accounted for 20%, 25%, and 17% of the variance in the model for late pregnancy-specific distress (for height, weight, and head circumference, respectively), 57% for late general distress, and 45% for late pregnancy-specific stress (PDQ). The second important keys were the relationships between mid- and late-maternal distress. Mid-general-pregnancy distress (index of perceived stress and depression/anxiety) had a significant relationship with late general-pregnancy distress. Additionally, mid-pregnancy-specific stress (PDQ) had a significant relationship with late pregnancy-specific stress. However, mid-maternal distress was not related directly to birth outcomes. The effect of mid-maternal distress on birth outcomes was related indirectly to late-maternal distress. The results showed that both late general distress (perceived stress, depression/anxiety) and late pregnancy-specific distress had direct negative effects on the three indexes of birth outcomes (weight, height, and head circumference). Table II shows that late perceived stress had a significant direct negative effect on the head circumferences, weight, and height of the neonates. In addition, late-depression/anxiety had an indirect effect on head circumferences, weight, and height of the neonates through late-pregnancy distress. An important pathway is the mediator role of late pregnancy-specific distress. The negative effect of late general-pregnancy distress and mid-pregnancy-specific distress on birth outcomes were mediated through late pregnancy-specific distress.

**Table I:** The comparison of mean and standard deviations of maternal distress in mid- and late pregnancy and the correlation between them

Maternal distress	Mid-pregnancy	Late-pregnancy	P-value	Correlation of Mid & Late r (p-value)
General Perceived Stress (PSS-14)	22.48░±░6.98	23.05░±░6.29	0.203	0.788 (< 0.001)
Pregnancy distress (PDQ)				
concerns about birth and the baby	10.28░±░5.17	10.39░±░5.19	0.775	0.727 (< 0.001)
concerns about weight/body image	5.10░±░1.86	4.92░±░2.58	0.494	0.337 (0.001)
concerns about emotions and relationships	2.68░±░2.05	2.76░±░1.98	0.671	0.567 (< 0.001)
Total PDQ	18.06░±░7.25	18.07░±░8.35	0.986	0.737 (< 0.001)
Depression and Anxiety (HADS)				
Depression	6.6░±░3.01	7.38░±░3.23	0.002	0.698 (< 0.001)
Anxiety	6.1░±░3.37	6.69░±░3.46	0.030	0.691 (< 0.001)
Total HADS	12.7░±░5.74	14.07░±░6.25	0.002	0.755 (< 0.001)

Data presented as mean░±░SD

Range scores: PSS-14; Perceived stress scale: 0–56, PDQ; Pregnancy distress questionnaire, concern about birth: 0–24; concern about weight and body image: 0–12; concerns about emotions and interpersonal relationships: 0–12, Total PDQ: 0–48. HADS; Hospital anxiety depression scale: anxiety, 0–21, depression: 0–21, Total scores HADS: 0–42

To compare the means between mid and late-pregnancy, t-test was used

To assess the association between scores of mid and late-pregnancy, Pearson correlation coefficient was used

**Table II:** Direct and indirect effect of the psychosocial predictors of birth outcomes

Psychosocial predictors		Effects		
		Direct	Indirect	Total effect
Perceived stress				
Mild				
	Height	-	-0.21	-0.21
	Weight	-	-0.26	-0.26
	Head circumference	-	-0.18	-0.18
Late				
	Height	-0.27	-0.06	-0.33
	Weight	-0.33	-0.06	-0.39
	Head circumference	-0.23	-0.06	-0.29
Depression/anxiety				
Mild				
	Height	-0.16	-	-0.16
	Weight	-0.18	-	-0.18
	Head circumference	-0.18	-	-0.18
Late				
	Height	-0.22	-	-0.22
	Weight	-0.24	-	-0.24
	Head circumference	-0.24	-	-0.24
Pregnancy distress				
Mild				
	Height	-	-0.07	-0.07
	Weight	-	-0.08	-0.08
	Head circumference	-	-0.08	-0.08
Late				
	Height	-	-0.1	-0.1
	Weight	-	-0.18	-0.18
	Head circumference	-	-0.18	-0.18

Data presented as correlation coefficient between variables

## 4 Discussion

The results showed that mid-maternal distress was not related directly to birth outcome. The effect of mid-maternal distress on birth outcome was related indirectly to late-maternal distress. Inconsistent with our results, a study reported that depression and anxiety in early pregnancy predicted higher levels of anxiety in late pregnancy (8). Previous research showed the significant relationship between late-maternal distress and preterm labor (12). Another report emphasized that early stress was a statistically significant determinant of mid-pregnancy specific-anxiety (13). women with stress and anxiety during pregnancy are at risk of having birth babies with lower APGAR scores (appearance, pulse, grimace, activity, and respiration) and lower psychomotor skills (14). In contrast with our results, some studies suggest that maternal stress during mid-pregnancy was significantly associated with poor neonatal and mental development of the infant (15, 16). Hassanjanzadeh and colleague concluded that maternal general stress is negatively associated with the head circumference. Additionally, depression symptoms during pregnancy were a negative predictor of APGAR scores of neonates (17).

The other main results of our path analysis confirmed that both late general-pregnancy stress (indexes of perceived stress and depression/anxiety) and late pregnancy-specific stress (PDQ) had direct negative effects on all of the indexes of birth outcome (weight, height, and head circumference). Inconsistent with our results, a study reported that pregnancy-specific distress in early, mid, and late pregnancy was associated negatively with birthweight (18).

The study has some limitations. First, we used some instruments to assess general or specific stress. Second, we could not conduct a continuous cohort evaluation for child development after birth. Third, the study employed correlational methods.

In conclusion, the results of the present study revealed that the negative effect of both mid-maternal distresses on birth outcome was related indirectly to late-maternal distress. Late-pregnancy distress has an indirect key role on negative birth outcomes. In addition, late pregnancy-specific distress mediates the relationships between late general-pregnancy distress (depression/anxiety and perceived stress), mid-pregnancy-specific stress, and birth outcome. This study provided a foundation for further research examining how pregnancy-specific distress, especially in late pregnancy, is a better predictor than pregnancy-general distress for the birth outcome.

## Conflict of Interest

The authors declare that there is no conflict of interest.
